# Comparative Analysis of 2-Point Jump Shot and Free Throw Kinematics in High- and Low-Level U18 Male Basketball Players

**DOI:** 10.3390/jfmk9040278

**Published:** 2024-12-19

**Authors:** Varvara Botsi, Dimitrios I. Bourdas, Antonios K. Travlos, Panteleimon Bakirtzoglou, Dimitrios C. Gofas, Ioannis E. Ktistakis, Emmanouil Zacharakis

**Affiliations:** 1 School of Physical Education and Sports Science, National and Kapodistrian University of Athens, 41 Ethnikis Antistasis, 17237 Daphne, Greece; varvarabotsi@gmail.com (V.B.); ktistakisy@phed.uoa.gr (I.E.K.); emzach@phed.uoa.gr (E.Z.); 2 Section of Sport Medicine & Biology of Exercise, School of Physical Education and Sports Science, National and Kapodistrian University of Athens, 41 Ethnikis Antistasis, 17237 Daphne, Greece; dbourdas@phed.uoa.gr; 3 Department of Sports Organization and Management, Faculty of Human Movement and Quality of Life Sciences, University of Peloponnese, Efstathiou and Stamatikis Valioti & Plataion Avenue, 23100 Sparta, Greece; atravlos@uop.gr; 4 School of Physical Education and Sport Science, Aristotle University of Thessaloniki, University Campus, 54124 Thessaloniki, Greece; 5 Arsakeia-Tositseia Schools, Philekpaideftiki Etaireia, Mitilinis 26, 11256 Athens, Greece; gofasd@hotmail.com

**Keywords:** dynamics, throwing, trajectory, angular velocity, entry angle, release time, performance, position, skill development, angular displacement

## Abstract

**Background/Objectives**: This study examined the influence of competition level and player position on shooting accuracy and kinematic parameters in U18 male basketball players, focusing on two-point jump shots and free throws. **Methods**: Thirty-eight higher-level (HL-group) and forty-one lower-level (LL-group) participants, categorized into guard, forward, and center subgroups, completed a two-point basketball shooting test, followed by a free-throw shooting test after a 30 min interval. These tests were administered using a crossover, counterbalanced approach with the Latin square method to ensure effective randomization. **Results**: The results indicated that the HL group displayed significantly faster (12.5%) shot release times (RTs) and closer-to-optimal 45° (8.1%) ball entry angles (EAs) into the hoop for free throws, as well as superior (24.2%) shot success rates (SSRs) for two-point jump shots compared with the LL group. Across all groups and subgroups, a higher EA was achieved in two-point shots than in free throws, though free throws showed higher SSR. This study found no positional differences in shooting mechanics or performance, suggesting that modern training practices may foster consistency across player roles. **Conclusions**: These findings emphasize the potential for targeted drills to improve RTs, EAs, and SSRs, especially in LL players. Coaches can apply these insights to enhance shooting mechanics and consistency, thereby elevating performance in young basketball athletes. Future research should investigate the impact of fatigue and defensive pressure on shooting parameters across varied competitive contexts.

## 1. Introduction

Shooting is a critical skill in basketball [1], with a team’s success highly dependent on effective scoring through free throws, two-point, and three-point shots (SSs) [2]. Among the various shooting techniques utilized in gameplay [3], the jump shot is particularly prominent, contributing to over 40% of total points in a typical game [4,5]. This technique involves the player propelling upward from the ground and releasing the ball near the peak of their jump, optimizing for both accuracy and range [6,7,8,9]. The jump shot requires a coordinated effort of the legs, core, and arms to generate the necessary force and control to direct the ball’s trajectory precisely toward the hoop [7,8,10]. This versatile shot is adaptable, allowing for variations in angle, release timing, and jump height based on the shooter’s position on the court and defensive pressures faced [7,8,11,12]. Similarly, the free throw (FT) also relies on a harmonized movement pattern involving the legs, torso, and arms to deliver both power and accuracy in guiding the ball toward the basket [7,8,13]. Thus, mastering both two-point jump shots and free throws demands extensive practice, particularly during childhood and adolescence, as numerous biomechanical and situational factors influence shot efficacy.

The release angle in basketball is widely considered a key factor influencing the success of both jump shots and free throws [14,15,16,17,18]. An increased release angle generally results in a higher ball trajectory, promoting a steeper entry angle (EA) as the ball approaches the hoop [12,16,17,18]. This sharper EA, which is closer to a perpendicular entry, effectively maximizes the usable rim area, enhancing the ball’s probability of passing through the hoop. Such an EA, however, demands a higher release velocity for precise execution [16,17,18,19]. The relationship between the release angle and EA is complex and influenced by several factors, including the shooter’s metabolic state [20,21], shooting distance [22], release height, and defensive pressures applied by opponents [5]. Empirical findings suggest that the ideal EA for a successful jump shot generally falls between 42° and 48°, with 45° being considered optimal [17,18,23]. However, the EA may vary among players of different ages and skill levels. For example, in elite male under-18 (U18M) players, an EA of approximately 41.38 ± 1.92° has been reported for jump shots from a 6.75 m distance [24]. Similarly, EAs of 32.30 ± 4.70° and 34.9 ± 5.9° have been observed for 2-point jump shots in elite male under-16 (U16M) and U18M players, respectively [19]. Among adult males (18.2 ± 2.2 y), EAs of 46.44 ± 3.17° for two-point jump shots at 5.80 m and 42.39 ± 1.85° for free throws have been documented [25]. Beyond the EA, the release time (RT) of a jump shot, defined as the interval from the moment a player receives the ball until it leaves their hand during the shooting action, is also critical for the speed and effectiveness of the shot. Studies of elite senior Serbian players have shown that RTs for a three-point jump shot range between approximately 0.76 and 0.83 s among guards, forwards, and centers, while RTs for a two-point jump shot vary between 1.07 and 1.23 s [11,15]. Additionally, U18M players have an RT of 0.80 ± 0.04 s for three-point shots [24], with RTs for two-point shots in elite U16M and U18M players measured at 0.84 ± 0.09 s and 0.84 ± 0.10 s, respectively [19]. Consequently, both EA and RT are essential kinematic factors influencing shot accuracy and overall shooting performance, shaping the effectiveness of jump shots and free throws [22,26].

Jump shots are utilized for both two-point and three-point scoring attempts. In EuroLeague games, teams typically attempt between 35 and 37 shots per game from the two-point range, covering a range of techniques, including layups, set shots, and jump shots [27]. In closely contested matches, even a single successful shot or free throw can significantly impact the outcome. Consequently, improving the success rates of two-point jump shots and free throws is critical for determining game results [27,28]. However, studies aimed at identifying optimal kinematic parameters—such as EA and RT—for three-point shots across different player positions remain limited [15,20] and are likely even scarcer for two-point shots and free throws. Research specifically examining EA and RT in two-point shots and/or free throws across various levels of under-18 male (U18M) basketball divisions and player positions is especially scarce, if not absent altogether.

While the significance of effective two-point and free-throw performance for achieving favorable outcomes across basketball competition levels is well established, there remains a gap in the literature regarding two-point jump shot and free throw accuracy, entry angles, release timing, and their potential associations with player positions within various divisions of national U18M basketball leagues. To address this gap in the literature, the current study aims to investigate two-point jump shot and free throw accuracy, entry angles, release timing, and their potential relationships to player positions across different league levels (i.e., higher-level versus lower-level) in U18M basketball. Our primary a priori hypotheses posit that both the player’s position and the division level will influence shooting accuracy and related kinematic factors for two-point jump shots and free throws. Additionally, this study seeks to identify any differences between two-point jump shots and free throws regarding accuracy and associated kinematic characteristics.

## 2. Materials and Methods

### 2.1. Subjects

**Figure 1:**
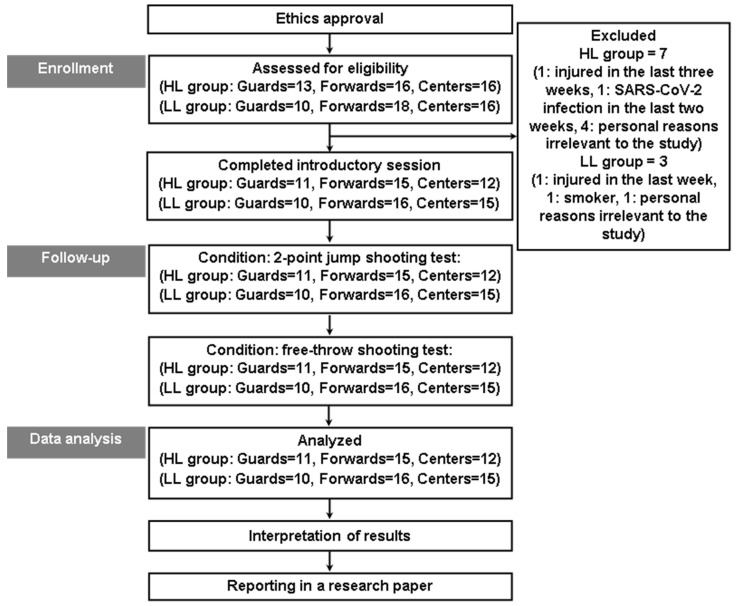
Schematic representation of this study’s experimental framework. Abbreviations: HL, higher-level; LL, lower-level.

This study’s experimental design received approval from the university’s ethics committee (Approval No: 1505/19-04-2023). Figure 1 provides a visual overview of the research framework. This study aimed to reach all registered adolescent basketball players in the Attica region. Eligibility criteria included athletes aged 16–18 who were physically active, defined as engaging in at least 60 min of moderate-to-vigorous physical activity per day [29]. Participants were required to have been free of musculoskeletal injuries for at least six months before the study and to exclusively play basketball. Exclusion criteria encompassed athletes with fewer than five years of basketball experience, visual or vestibular impairments, insufficient sleep (less than 8 h daily), or any severe health conditions. Additionally, athletes under active medication with a history of smoking or alcohol use were not eligible to participate. Parents or guardians completed screening questionnaires covering physical activity and sleep [30,31,32,33], and all participants underwent a medical examination. A total of 79 basketball players were evaluated for this study. Of these, 38 athletes were members of teams competing in the Greek A league for adolescents (U18), classified as the higher-level (HL) group, while 41 athletes competed in the B league, forming the lower-level (LL) group. Each group was further subdivided by playing position into guards, forwards, and centers. Detailed anthropometric and physiological data for each subgroup are presented in Table 1. All participants and their parents provided written informed consent after receiving comprehensive verbal and written explanations of this study’s purpose and methods [34]. Participants were assured of their right to withdraw at any time without providing a reason, in alignment with the Helsinki Declaration [34].

### 2.2. Initial Assessments

**Figure 2:**
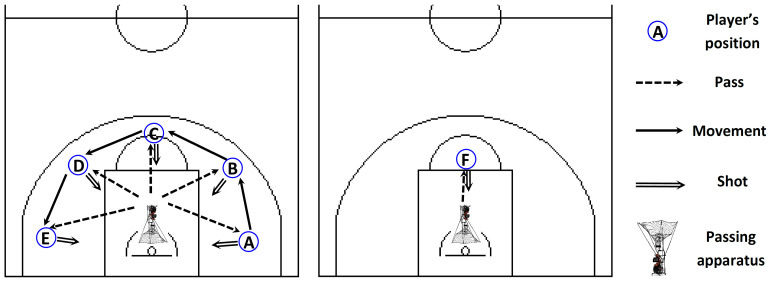
Two-point jump shooting test (**left**). The participants, from a 5.57 m distance [37], performed a sequence of five consecutive two-point jump shots from the A to E positions (i.e., left corner, left wing, top, right wing, and right corner), with twenty-five shots in total. Free throw shooting test (**right**). The participants performed five consecutive free-throw shots from the free-throw line (F position).

At the start of the study, during their initial visit to the research facility, all participants were thoroughly acquainted with the laboratory and court-field environments. They received an in-depth briefing on the research methodologies and procedures to be used throughout the study. Subsequently, measurements were taken for height (Stadiometer^®^, Seca, Birmingham, UK), sitting height and leg length (Height Indicator Tape Ruler, Posh Rulers, OR, USA), and body mass (Beam Balance 710, Seca, Birmingham, UK). Maturity offset was then calculated according to the formula proposed by Mirwald et al. [35]: maturity offset = −9.236 + 0.0002708·(leg length × sitting height) − 0.001663·(age × leg length) + 0.007216·(age × sitting height) + 0.02292·(weight/height ratio). Handgrip strength was assessed using a dynamometer (Takei T.K.K.5401 GRIP-D handgrip dynamometer, Takei Scientific Instruments Co., Ltd., Tokyo, Japan) following the methodology outlined by Gatt et al. [36]. Participants were then introduced to the basketball shooting protocols, including the 2-point jump shooting test [37] and a free-throw shooting test, as shown in Figure 2.

### 2.3. Methodological Protocols

Seven days later, at the second visit, participants completed a 2-point basketball shooting test (2-point condition), followed by a free throw shooting test (free throw condition) after a 30 min interval. These tests were administered using a crossover, counterbalanced approach with the Latin square method to ensure effective randomization. Testing was conducted on a standard indoor hardwood basketball court, where participants were instructed to exert maximum effort as they would during regular practice sessions.

For the two days leading up to both the initial and subsequent visits (in both laboratory and court settings), participants adhered to a regimen that avoided intensive physical exertion, limiting activity to post-game recovery sessions focused on reinforcing gameplay strategies and team dynamics [38,39]. To maintain consistency, participants abstained from any supplements with potential ergogenic or synergistic effects, following established research findings [40,41]. Additionally, participants were provided with a standardized dinner the evening prior to each experimental session. Before the second visit, participants arrived at the court between 10:00 and 10:30 a.m., having fasted overnight. The court conditions were kept stable, with an ambient temperature of 24–25 °C, barometric pressure between 1005 and 1035 mmHg, and relative humidity of around 50%.

Upon arrival, participants were instructed to empty their bladders before beginning any experimental tasks. They then performed a standardized 20 min warm-up, including light jogging, dynamic stretching, short bursts of high-intensity running, and fifty practice shots (either two-point jump shots or free throws, depending on the test condition). Prior to each test, all measurement equipment was calibrated according to the manufacturer’s guidelines to ensure accuracy.

Participants were kept unaware of their performance scores to prevent bias and were asked not to discuss the study with others to avoid preconceived expectations. Additionally, both participants and assistant researchers were blinded to the true aim of this study. This study took place in November during the early phase of the regular season, when players were expected to have relatively low levels of cumulative fatigue due to the limited number of games played at that point in the season [42,43]. All participants were in good health, reported feeling well, and avoided beverages or medications containing caffeine or alcohol throughout the study.

### 2.4. Basketball Shooting Tests and Kinematic Parameter Measurements

For the two-point jump shooting assessment, participants adopted an offensive or “triple-threat” stance, involving flexed ankle, knee, and hip joints, direct gaze at the hoop, shoulder-width foot placement, and both feet grounded. This assessment demonstrated high reliability (r = 0.89) and validity (r = 0.83), as reported by the American Alliance for Health Physical Education Recreation and Dance [37,44]. Each participant performed five consecutive jump shots from five distinct positions on the court (left corner, left wing, center, right wing, and right corner) for a total of 25 shots (Figure 2). These shots were taken without any defensive interference, positioned 5.57 m from the basket in alignment with the age-specific guidelines [37]. Participants were asked to shoot with their dominant hand as they would during an actual game, aiming for a swish, and were allowed a 150 s time limit to complete all 25 attempts. To facilitate this process, smart sensor basketballs (94Fifty Basketball, InfoMotion Sports Technologies Inc., Dublin, OH, USA) and a passing machine (Dr. Dish CT+, Airborne Athletics, Inc., Minneapolis, MN, USA) were employed to deliver the ball consistently at chest height with regular timing. The passing machine enabled accurate tracking of each shot attempt (SS), allowing us to calculate shooting accuracy and derive the shooting success rate (SSR) for analysis. The smart basketballs used were regulation size and weight, featuring nine embedded pressure sensors, a Bluetooth module, and a rechargeable battery with an eight-hour lifespan. These sensors provided a full 360-degree analysis of ball handling forces, allowing for detailed tracking of shooting metrics. Real-time data transmission to the 94Fifty app (Galaxy-A705FN/DS, Samsung, Suwon, Republic of Korea) occurred within 100 milliseconds and covered a range up to 30 m. Past studies have confirmed the accuracy and consistency of these kinematic measurements (e.g., entry angle (EA) and release time (RT)), with a reliability index of α = 0.998 compared with video analysis [45,46]. For the free-throw shooting test, participants also maintained the offensive stance and executed five consecutive free throws with their dominant hand under a 30 s time limit, simulating real-game conditions (Figure 2). Again, smart sensor basketballs and the Dr. Dish CT+ passing machine were used to facilitate shot collection and delivery with consistent intensity and timing.

This setup allowed us to calculate the basketball’s EA, RT, SS, and SSR. Additionally, a camera (Hero 9, GoPro Inc., San Mateo, CL, USA) was used to record the trials, and two external experts reviewed the footage to confirm uniform shooting techniques across participants [7]. Heart rate (HR) data were continuously recorded at 5 s intervals using a Polar RCX5 telemetric device (Polar Electro Oy, Kempele, Finland) throughout the tests, enabling calculations of average HR before and after each shooting trial. Participants also reported their subjective rating of perceived exertion (RPE) using the 6–20 Borg scale [47], both before and after the tests. Heart rate and RPE data were collected as physiological indicators of the players’ responses to the internal load of shooting tests.

### 2.5. Statistical Analyses

The dataset was assessed for normality and homogeneity of variances using Shapiro–Wilk and Levene’s tests, respectively, and met the required assumptions for both (*p* > 0.05). For the two groups categorized by league levels (higher-level and lower-level groups), one-way analysis of variance (ANOVA) was used to evaluate differences in participant characteristics as well as mean values for RT, EA, SS, and SSR for each shooting test type (two-point and free throw) across different playing positions (guards, forwards, and centers). When significant differences emerged, Tukey’s post hoc test was used to determine specific pairwise differences [48].

Independent *t*-tests were performed to compare the mean RT, EA, SS, and SSR between each position subgroup within the two groups (e.g., HL guards vs. LL guards) as well as overall between the higher-level and lower-level groups for each shooting test type [48]. Additionally, paired *t*-tests were conducted to compare these variables between the two shooting conditions (two-point and free throw) within the same position subgroups and overall within each group [48].

To evaluate differences in the HR and RPE adjusted post-test means between the higher-level and lower-level groups, as well as across the different playing positions, one-way analysis of covariance (ANCOVA) was conducted, with pre-test values used as covariates. When significant ANCOVA results were obtained, Bonferroni-corrected pairwise comparisons were utilized for post hoc analysis [48]. All statistical analyses were conducted using IBM SPSS Statistics software, version 29.0 (IBM Corp., Armonk, NY, USA), with the significance level set at α = 0.05 for all tests.

## 3. Results

Data are presented as means ± standard deviations (SDs) with 95% confidence intervals (CIs) shown in square brackets, unless otherwise indicated. The accuracy metrics and associated kinematic variables of both two-point jump shots and free throws, organized by player position for each group, are summarized in Table 2.

Significant group differences emerged: the release time for free throws was notably faster in the HL group compared with that in the LL group (t(77) = −3.213, *p* = 0.002). Furthermore, the entry angle for two-point jump shots was greater in the HL group than in the LL group (t(77) = 2.856, *p* = 0.006), and the shooting success rate for two-point jump shots was also significantly higher in the HL group (t(77) = 3.137, *p* = 0.002).

A comparison between shot types revealed that the EA during two-point jump shots was significantly greater than that during free throws for both HL (t(37) = 10.472, *p* < 0.001) and LL (t(40) = 25.621, *p* < 0.001) groups, as well as across all positional subgroups, including HL guards (t(10) = 6.662, *p* < 0.001), HL forwards (t(14) = 6.074, *p* < 0.001), HL centers (t(11) = 5.435, *p* < 0.001), LL guards (t(9) = 5.071, *p* = 0.001), LL forwards (t(15) = 15.348, *p* < 0.001), and LL centers (t(14) = 14.247, *p* < 0.001).

In terms of SSR, free throws showed a significantly higher success rate than two-point jump shots in both HL (t(37) = −2.695, *p* = 0.011) and LL (t(40) = −3.965, *p* < 0.001) groups. This trend was consistent across all positional subgroups, though the difference reached statistical significance only within HL centers (t(11) = −3.391, *p* = 0.006), LL forwards (t(15) = −2.648, *p* = 0.018), and LL centers (t(14) = −2.691, *p* = 0.018).

No significant associations were observed between player position and accuracy, EA, or RT in either two-point jump shots or free throws. Additionally, Table 3 presents the estimated heart rate and ratings of perceived exertion, adjusted for pre-shooting values, reflecting internal load responses at the conclusion of each shooting trial (two-point jump shots and free throws) across groups and positional subgroups.

## 4. Discussion

The primary objective of this study was to investigate two-point jump shooting and free throw accuracy, along with their associated kinematic parameters, in relation to player positions and team division levels within U18M basketball. Our findings reveal several statistically significant differences across groups. Specifically, the higher-level (HL) group exhibited a faster release time (RT) in free throws, a more optimal entry angle (EA, closer to the ideal 45°) in both free throws and two-point jump shots, and an elevated shooting success rate (SSR) in two-point jump shots compared with the lower-level (LL) group. Additionally, in both groups and across all positional subgroups, the EA during two-point jump shots was consistently higher and closer to the optimal angle compared with free throws. A higher SSR was also observed for free throws relative to two-point jump shots, particularly in HL centers, LL forwards, and LL centers. While these results highlight meaningful differences between groups and shot types, it is essential to view these findings as preliminary, providing a foundation for further exploration into how division level and player positions influence shooting performance and kinematic characteristics in youth basketball.

Modern basketball is recognized as a dynamic team sport [49] that emphasizes a strong command of technical skills to excel in competition [3,50,51]. Among these essential skills, jump shots and free throws play a pivotal role, as they contribute a substantial proportion of the game’s total points—jump shots alone can account for over 40% of points scored in a match [4,5]. Since basketball ultimately rewards the team with the highest score [2], accurate shooting techniques, such as achieving the optimal entry angle around 45° [17], and swift release times (e.g., RT ≤ 0.65 s in skilled players), become critical to avoid defensive interference. Previous studies involving elite players using similar smart basketball technology identified a typical entry angle and release time of approximately 43.5 ± 1.7° [43.0–44.1] and 0.7 ± 0.1 s [0.7–0.7], respectively, for three-point jump shots in elite adult players [20], while slightly lower entry angle values were observed in 18–19-year-old guards and forward centers (EA: 41.5 ± 3.1° for guards and 41.6 ± 3.0° for forward centers; RT: 0.8 ± 0.1 s for guards, 0.8 ± 0.1 s for forward centers) [15]. Interestingly, earlier research noted that jump shots and free throws often exhibited similar release angles and speeds, suggesting a tendency toward consistent motor patterns across shooting types [52]. This similarity may be attributable to the mental fixation on release mechanics in both shooting forms or to the minimal difference in release height, which does not necessitate a significant speed adjustment. Further, a study on two-point jump shots from 5.8 m and free throws using similar technology indicated an entry angle of 46.4 ± 3.2° for two-point shots and 42.4 ± 1.8° for free throws among 18.0 ± 2.2-year-old players [25]. Consistent with this, our findings showed a more favorable entry angle—closer to the optimal 45°—for two-point shots than for free throws across all groups and subgroups, although SSR was generally higher for free throws compared with two-point shots, as anticipated [53]. This paradox is intriguing, as one might expect shot mechanics to align more consistently with outcomes. Several factors may contribute to this disparity. For adolescents, whose muscle strength, coordination, and motor control are still developing, the forceful mechanics of a two-point jump shot might naturally produce a higher arc [53,54,55]. In contrast, free throws generally require a more controlled release, which could yield a flatter trajectory [21,25]. Furthermore, the mental pressure and isolated nature of free throws may cause players to overthink their form, potentially disrupting the natural shooting arc [56]. Though U18 players likely have a solid grasp of fundamental basketball skills, their technique and consistency in adapting shot angles for specific scenarios may improve with experience. While these explanations are plausible, individual player differences, coaching methods, and training regimens also play substantial roles in shaping shooting mechanics and performance. Further research is required to clarify these influences, which could enhance our understanding of shot arc variations and success rates in developing players.

A recent study on three-point jump shooting in high-level adult male basketball players demonstrated that shooting accuracy, release time, and ball entry angle can vary across positions [20]. In contrast, previous findings from high-level adolescent male players indicated no significant positional differences in release time, ball rotation, or entry angle between guards, forwards, and centers during three-point shots [15]. Our study similarly revealed no positional differences, despite significant distinctions between skill levels (high-level vs. low-level), which warrants further exploration. The consistency in heart rate and rating of perceived exertion at the conclusion of the shooting tests (two-point and free throw) across groups and subgroups—adjusted for pre-shooting baselines—suggests that internal load indicators (e.g., cardiac response, muscular exertion, and fatigue) remained stable, showing no substantial impact on shooting outcomes across skill levels [57]. A likely contributor to these findings is the role of coaching quality and training exposure in influencing shooting mechanics and performance. When players receive similar coaching and training appropriate to their developmental stage, positional differences may be reduced. Moreover, in modern basketball, young athletes increasingly develop versatile skill sets, including shooting proficiency across positions [3,49,50,51], which could contribute to the reduced variability in shooting mechanics by position [15]. Additionally, contemporary youth development programs tend to emphasize a strong foundation in fundamental skills, particularly shooting, for all players, regardless of their designated positions [58], promoting more homogeneous skill development. To better understand the intricate relationships between position, skill level, and shooting performance, future research should consider: (i) conducting detailed performance analyses to identify factors such as release angle, ball spin rate, and landing position, that may influence shooting accuracy; (ii) applying qualitative video analysis to explore positional shooting mechanics in depth; and (iii) interviewing coaches and players to gain insights into training methodologies, player development pathways, and the evolving expectations of player versatility across basketball positions.

It was hypothesized that players competing at a higher division level in basketball would demonstrate superior shooting mechanics [59], characterized by faster release times, optimal shot arcs, and increased shooting accuracy [15,20,25,60]. This hypothesis is supported by our findings, which show that the HL group exhibited significantly faster release times for free throws, a more optimal entry angle (closer to the ideal 45°) for free throws, and a higher shooting success rate for two-point jump shots compared with the LL group. While these differences align with expectations given the enhanced training, coaching, and experience typically associated with higher-level teams [59,60], the novelty of our findings lies in identifying and quantifying the specific kinematic variables—release time, entry angle, and shooting success rate—where these disparities are most evident. These findings provide actionable insights for coaches aiming to develop these critical performance indicators in lower-level athletes. Moreover, they contribute to the growing body of research emphasizing the importance of biomechanical and situational factors in basketball shooting performance [61,62,63,64,65]. Future research should further explore how these variables evolve with player development and investigate additional factors, such as psychological resilience and decision-making, that may differentiate skill levels.

### Limitations, Strengths, and Suggestions for Future Research and Practical Applications

This study, while offering important insights, comes with several limitations that are crucial to acknowledge. Firstly, our participant pool consisted solely of U18 male athletes, which may limit the generalizability of the findings across different age groups, genders, and competitive levels. Secondly, shooting tests were conducted without preceding physical exertion and therefore did not fully emulate the fatigue dynamics present over a typical 40 min basketball game. This controlled setting omits potential fatigue-related changes in physical, physiological, and psychological factors that could significantly impact shooting performance under actual game conditions. Additionally, our analysis was constrained by the lack of data on other detailed kinematic variables, such as joint positions, angular velocities, and hip displacement [66], which might further inform the biomechanical nuances of shooting performance. Furthermore, while this study focused on two-point jump shots and free throws, it did not include three-point jump shots, which are becoming increasingly critical in modern basketball, particularly at higher competitive levels such as the EuroLeague, US college basketball, and the NBA [27,28,67]. This omission is a notable limitation as three-pointers require distinct shooting mechanics and decision-making processes that could offer additional insights. Finally, the absence of defensive pressure during shooting does not fully simulate in-game scenarios [5].

Despite these limitations, this study employs advanced technology and rigorous methodology, building upon prior findings regarding shot accuracy, release time, and entry angle in relation to player position across competitive levels. This study highlights differences between higher- and lower-level athletes that warrant further research to deepen our understanding of shooting mechanics and accuracy in two-point jump shots and free throws. Future investigations would benefit from the inclusion of diverse, game-like protocols or competitive practice settings to evaluate the impact of fatigue on shooting form across various shot types, distances, genders, ages, and competitive levels in basketball players. Moreover, while this study focuses on the influence of training and competition level on shooting mechanics and accuracy, future research could explore the potential role of innate factors, such as genetic predispositions or inherent motor abilities, in determining shooting performance. Such investigations could help to disentangle the contributions of natural aptitude versus training in basketball skill development.

The findings provide valuable implications for coaches and practitioners working to enhance shooting proficiency among youth basketball athletes [68]. The observed differences in release timing, entry angle, and shooting success rate between HL and LL groups underscore the value of customized training interventions based on skill level. Coaches may prioritize drills for LL athletes that emphasize faster release and the attainment of an optimal shot arc (close to 45°) to improve accuracy. Although no positional differences were identified, coaches should continue to develop shooting skills uniformly across player positions, supporting the trend toward positionless basketball and building versatile shooters across the roster. The more effective entry angles observed in HL players reinforce the critical role of shot trajectory in maximizing shooting success. By using video feedback and sensor-based data, coaches can aid players in understanding and achieving a more effective arc, fostering muscle memory for an optimal shot trajectory, particularly for LL players. Additionally, the quicker release times among HL athletes suggest a beneficial training focus on game-speed shooting for LL players, with defensive pressure and time constraints to improve reaction time and accuracy under competitive conditions. Finally, this study’s finding of higher SSRs for free throws relative to two-point jump shots in both skill levels, combined with the known correlation between reduced entry angle and lower free throw success [69], emphasizes the potential for SSR improvement in both groups. Targeted free throw training with an emphasis on achieving a higher entry angle may yield better SSR outcomes. Separate and focused training of two-point shots and free throws, alongside adjustments in angle and release mechanics, could contribute meaningfully to shooting proficiency.

## 5. Conclusions

This study provides a comprehensive analysis of 2-point jump shots and free throws among U18 male basketball players, comparing shooting accuracy and associated kinematic parameters between groups defined by league levels (higher-level and lower-level) and player positions. The results indicated that players in the higher-level group exhibited statistically faster release times for free throws, achieved entry angles closer to the optimal 45° for enhanced shooting accuracy, and demonstrated a higher shooting success rate in two-point jump shots compared with their counterparts in the lower-level group. While player position did not yield significant differences in shooting mechanics, the findings suggest that league level, which likely reflects differences in player experience and training, plays a pivotal role in refining key performance indicators, such as release time and entry angle. These findings underscore the critical impact of league level on shooting mechanics and highlight the need for tailored training to address specific skill gaps in lower-level players, particularly regarding shot speed and entry angle consistency. It is important to note that the findings of this study are specific to U18 male athletes and may not fully generalize to other age groups, genders, or league levels. This study’s findings suggest several practical applications for coaches and practitioners. These include prioritizing drills for lower-level players to improve release speed and achieve an optimal shot arc, using video feedback and sensor-based data to enhance shooting mechanics, and focusing on game-speed shooting practices under defensive pressure to simulate competitive scenarios. Emphasizing uniform shooting skill development across positions aligns with the growing trend of positionless basketball and prepares players for diverse roles on the court. Nonetheless, the results also suggest that young basketball players, regardless of position, benefit from versatile shooting skill development, supporting the trend toward a positionless style of play in modern basketball.

## Figures and Tables

**Table 1:** Comprehensive analysis of anthropometric and physiological characteristics in guard, forward, and center subgroups, and overall groups (M ± SD [95% CI]).

Group/ Subgroup	Height (cm)	Body Mass (kg)	BMI (kg·m^−2^)	Age (y)	Handgrip Strength (kg)	Maturity Offset (y)	Basketball Experience (y)	Basketball Training (h·wk^−1^)	Physical Exercise (h·wk^−1^)
HL overall (n = 38)	184.3 ± 6.5 [182.2–186.4]	74.2 ± 10.0 [71.0–77.4]	21.8 ± 2.6 [21.0–22.6]	16.7 ± 0.8 [16.4–17.0]	44.7 ± 8.0 [42.2–47.2]	* 2.3 ± 0.7 [2.1–2.5]	8.3 ± 2 [7.7–8.9]	* 10.2 ± 4.0 [8.9–11.5]	2.1 ± 1.3 [1.7–2.5]
HL-guards (N = 11)	^†^ 181.4 ± 5.9 [177.9–184.9]	73 ± 8.7 [67.9–78.1]	22.2 ± 2.1 [21.0–23.4]	16.6 ± 0.7 [16.2–17]	45.3 ± 8.6 [40.2–50.4]	2.0 ± 0.9 [1.5–2.5]	8.6 ± 2.3 [7.2–10.0]	10.8 ± 5.0 [7.8–13.8]	2.1 ± 1.3 [1.3–2.9]
HL-forwards (N = 15)	^‡^ 181.9 ± 4.7 [179.5–184.3]	^‡^ 69.1 ± 10.4 [63.8–74.4]	20.9 ± 3.2 [19.3–22.5]	16.7 ± 1.1 [16.1–17.3]	43.0 ± 8.0 [39.0–47]	** 2.2 ± 0.6 [1.9–2.5]	8.7 ± 1.9 [7.7–9.7]	** 10.0 ± 3.3 [8.3–11.7]	1.9 ± 1.5 [1.1–2.7]
HL-centers (N = 12)	189.9 ± 5.6 [186.7–193.1]	81.7 ± 5.4 [78.6–84.8]	22.7 ± 1.8 [21.7–23.7]	16.8 ± 0.6 [16.5–17.1]	46.3 ± 7.7 [41.9–50.7]	** 2.5 ± 0.4 [2.3–2.7]	7.4 ± 1.7 [6.4–8.4]	** 10.0 ± 4.2 [7.6–12.4]	2.5 ± 0.8 [2.0–3.0]
LL overall (n = 41)	181.7 ± 6.4 [179.7–183.7]	75.6 ± 12.6 [71.7–79.5]	22.9 ± 3.6 [21.8–24.0]	16.6 ± 0.7 [16.4–16.8]	41.1 ± 6.6 [39.1–43.1]	1.7 ± 0.7 [1.5–1.9]	8.9 ± 2.6 [8.1–9.7]	8.1 ± 3.2 [7.1–9.1]	1.4 ± 2.2 [0.7–2.1]
LL-guards (N = 10)	^†^ 179.8 ± 6 [176.1–183.5]	^†^ 68.2 ± 4.1 [65.7–70.7]	^†^ 21.2 ± 2.2 [19.8–22.6]	16.6 ± 0.7 [16.2–17.0]	42.2 ± 6.0 [38.5–45.9]	1.6 ± 0.7 [1.2–2]	9.6 ± 2.7 [7.9–11.3]	10.2 ± 4.0 [7.7–12.7]	1.8 ± 2.0 [0.6–3.0]
LL-forwards (N = 16)	^‡^ 178.4 ± 6 [175.5–181.3]	^‡^ 70.2 ± 4.5 [68.0–72.4]	22.1 ± 1.9 [21.2–23.0]	16.6 ± 0.7 [16.3–16.9]	37.8 ± 6.7 [34.5–41.1]	1.5 ± 0.7 [1.2–1.8]	9.1 ± 2.4 [7.9–10.3]	7.6 ± 2.9 [6.2–9]	1.1 ± 1.9 [0.2–2.0]
LL-centers (N = 15)	186.5 ± 4.1 [184.4–188.6]	86.3 ± 15.1 [78.7–93.9]	24.9 ± 4.8 [22.5–27.3]	16.5 ± 0.7 [16.1–16.9]	43.8 ± 5.7 [40.9–46.7]	2.1 ± 0.7 [1.7–2.5]	8.2 ± 2.8 [6.8–9.6]	7.2 ± 2.5 [5.9–8.5]	1.5 ± 2.6 [0.2–2.8]

* Significant difference between HL and LL groups overall at *p* ≤ 0.05. ** Significant difference between subgroups at *p* ≤ 0.05. ^†^ Significant difference between guard and center subgroups in the same group at *p* ≤ 0.05. ^‡^ Significant difference between forward and center subgroups in the same group at *p* ≤ 0.05. Abbreviations: BMI, body mass index; HL, higher-level athletes; LL, lower-level athletes; M, mean; n, sample size of the study groups; N, sample size of the subgroups; SD, standard deviation; and [95%CI], 95% confidence interval.

**Table 2:** The M ± SD [95%CI] of the shot release time, ball’s entry angle into the hoop, and shooting success rates for higher- and lower-level athlete groups overall, and by guard, forward, and center subgroups during the two-point jump shooting and free throw tests.

	2-Point Jump Shooting Test			Free Throw Test		
Group/ Subgroup	RT (s)	EA (°)	SSR (%)	RT (s)	EA (°)	SSR (%)
HL overall (n = 38)	0.8 ± 0.4 [0.7–0.9]	^¶^ 42.5 ± 4.3 [41.1–43.9]	*^¶^ 45.4 ± 15.4 [40.5–50.3]	* 0.7 ± 0.1 [0.7–0.7]	* 35.9 ± 4.3 [34.5–37.3]	57.4 ± 21.4 [50.6–64.2]
HL guards (N = 11)	0.7 ± 0.1 [0.6–0.8]	^¶^ 44.1 ± 5.6 [40.8–47.4]	^§^ 47.6 ± 14.1 [39.3–55.9]	^§^ 0.7 ± 0.2 [0.6–0.8]	37.3 ± 4.2 [34.8–39.8]	47.3 ± 16.2 [37.7–56.9]
HL forwards (N = 15)	0.7 ± 0.1 [0.6–0.8]	^¶^ 43.1 ± 3.1 [41.5–44.7]	48.8 ± 14.5 [41.5–56.1]	0.7 ± 0.1 [0.6–0.8]	36.1 ± 4.6 [33.8–38.4]	58.7 ± 24.5 [46.3–71.1]
HL centers (N = 12)	0.9 ± 0.7 [0.5–1.3]	^¶^ 40.3 ± 3.6 [38.3–42.3]	^¶^ 39.0 ± 16.9 [29.4–48.6]	0.7 ± 0.1 [0.6–0.8]	34.4 ± 3.7 [32.3–36.5]	65.0 ± 19.3 [54.1–75.9]
LL overall (n = 41)	0.9 ± 0.8 [0.7–1.1]	^¶^ 41.7 ± 5.0 [40.2–43.2]	^¶^ 34.4 ± 15.5 [29.7–39.1]	0.8 ± 0.2 [0.7–0.9]	33.0 ± 4.8 [31.5–34.5]	50.7 ± 25.7 [42.8–58.6]
LL guards (N = 10)	1.3 ± 1.6 [0.3–2.3]	^¶^ 43.1 ± 3.1 [41.2–45.0]	33.6 ± 14.3 [24.7–42.5]	0.9 ± 0.1 [0.8–1.0]	33.9 ± 3.5 [31.7–36.1]	48.0 ± 28.6 [30.3–65.7]
LL forwards (N = 16)	0.7 ± 0.1 [0.7–0.7]	^¶^ 42.4 ± 5.0 [40.0–44.8]	^¶^ 40.5 ± 17.8 [31.8–49.2]	0.8 ± 0.2 [0.7–0.9]	33.6 ± 4.4 [31.4–35.8]	53.8 ± 27.0 [40.6–67]
LL centers (N = 15)	0.7 ± 0.1 [0.6–0.8]	^¶^ 40.1 ± 5.8 [37.2–43]	^¶^ 28.5 ± 11.8 [22.5–34.5]	0.8 ± 0.2 [0.7–0.9]	31.7 ± 6.0 [28.7–34.7]	49.3 ± 23.7 [37.3–61.3]

* Significant difference between HL and LL groups overall at *p* ≤ 0.05. ^§^ Significant difference between HL guards and LL guards subgroups at *p* ≤ 0.05. ^¶^ Significant difference between two-point jump shooting and free throw test variables in the same group/subgroup at *p* ≤ 0.05. Abbreviations: EA, entry angle into the hoop; HL, higher-level athletes; LL, lower-level athletes; M, mean; n, sample size of the study groups; N, sample size of the subgroups; RT, shot release time; SD, standard deviation; SSR, shooting success rate; and [95%CI], 95% confidence interval.

**Table 3:** Estimates ^a^ of heart rate and subjective rating of perceived exertion at the end of shooting trials (i.e., two-point jump shooting and free throw) by group and subgroup, adjusted for pre-shooting covariate values.

	Two-Point Jump Shooting Test		Free Throw Test	
**Group**	^1^ **HR (b · min^−1^)**	^3^ **RPE (Borg scale, 6–20)**	^5^ **HR (b · min^−1^)**	^7^ **RPE (Borg scale, 6–20)**
HL overall (n = 38)	133.6 ± 0.1 [133.5–133.7]	6.7 ± 0.1 [6.6–6.9]	95.4 ± 0.1 [95.3–95.6]	6.5 ± 0.1 [6.4–6.6]
LL overall (n = 41)	133.6 ± 0.1 [133.6–133.7]	6.7 ± 0.1 [6.5–6.8]	95.6 ± 0.1 [95.4–95.7]	6.5 ± 0.1 [6.4–6.6]
**Subgroup**	^2^ **HR (b·min^−1^)**	^4^ **RPE (Borg scale, 6–20)**	^6^ **HR (b·min^−1^)**	^8^ **RPE (Borg scale, 6–20)**
Guards overall (N = 21)	133.7 ± 0.1 [133.6–133.8]	6.8 ± 0.1 [6.6–7.0]	95.3 ± 0.1 [95.2–95.5]	6.5 ± 0.1 [6.4–6.7]
Forwards overall (N = 31)	133.6 ± 0.1 [133.5–133.7]	6.7 ± 0.1 [6.5–6.9]	95.5 ± 0.1 [95.4–95.7]	6.5 ± 0.1 [6.4–6.6]
Centers overall (N = 27)	133.6 ± 0.1 [133.5–133.7]	6.7 ± 0.1 [6.510–6.8]	95.5 ± 0.1 [95.4–95.7]	6.5 ± 0.1 [6.4–6.6]

^a^ Estimates are presented as the mean ± standard error [95% confidence interval]; Covariates appearing in the model are evaluated at the following pre-shooting values, respectively: ^1^ 66.80, ^2^ 66.85, ^3^ 6.46, ^4^ 6.46, ^5^ 66.80, ^6^ 66.85, ^7^ 6.46, ^8^ 6.46. Abbreviations: HL, higher-level athletes; HR, heart rate; LL, lower-level athletes; n, sample size of the study groups; N, sample size of the subgroups; and RPE, subjective rating of perceived exertion.

## Data Availability

The raw data supporting the conclusions of this article will be made available by the corresponding author upon reasonable request once all relevant substudies are reported and completed. The study protocol, data dictionary, and statistical analysis plan can also be made available by the corresponding author upon request.
